# Supplementary material for: Acute Kidney Injury after Endovascular Treatment in Patients with Acute Ischemic Stroke
Source: J Clin Med. 2020 May 14;9(5):1471. doi: 10.3390/jcm9051471 (PMC7291207; doi:10.3390/jcm9051471)
Supplement: Supplementary file 1 [file jcm-09-01471-s001.pdf]

Table S1. Characteristics of included and excluded patient.

	Included patient (n = 601)	Excluded patient (n = 98)	p-value
Age, years	68.0 ± 12.2	63.8 ± 12.6	0.003
Sex, men	333 (55.4)	53 (54.1)	0.892
Risk factors			
Hypertension	381 (63.4)	52 (53.1)	0.066
Diabetes mellitus	167 (27.8)	22 (22.4)	0.327
Atrial fibrillation	298 (49.6)	47 (48.0)	0.850
Dyslipidemia	180 (30.0)	25 (25.5)	0.438
Smoking	128 (21.3)	34 (34.7)	0.005
Previous stroke or history of TIA	107 (17.8)	13 (13.3)	0.337
Baseline renal function			0.003 *
eGFR ≥90 mL/min/1.73 m^2	98 (16.3)	20 (21.1)	
eGFR 60–89 mL/min/1.73 m^2	211 (35.1)	45 (47.4)	
eGFR 30–59 mL/min/1.73 m^2	252 (41.9)	29 (30.5)	
eGFR <30 mL/min/1.73 m^2	40 (6.7)	1 (1.0)	
eGFR	64.6 ± 26.1	72.4 ± 24.6	0.005
Contrast agent			0.462
Iodixanol	435 (72.4)	75 (76.5)	
Iopamidol	166 (27.6)	23 (23.5)	
Contrast dose, mL	71.2 ± 37.2	66.4 ± 32.6	0.194
Laboratory findings			
Hemoglobin, g/dL	13.4 ± 1.9	13.7 ± 1.8	0.207
White blood cell count, ×10^9/L	8.8 ± 3.5	8.5 ± 2.5	0.238
Platelets, ×10^9/L	221 ± 69	228 ± 64	0.319
Glucose, mmol/L	7.8 ± 3.1	7.5 ± 2.6	0.300
Stroke-related factors			
NIHSS score on admission	17 (13–21)	14 (9–18)	<0.001
Intravenous tPA	310 (51.6)	54 (55.1)	0.591
Onset to puncture time, min	269 (180–437)	244 (165–422)	0.211
Procedure time, min	62 (43–90)	46 (34–81)	<0.001
Unsuccessful reperfusion (mTICI 2a or less)	149 (24.8)	11 (11.2)	0.005
Outcomes			
mRS at 3 months	3 (1–5)	1 (0–2)	<0.001
Good functional outcome (mRS 0–2)	277 (46.1)	84 (85.7)	<0.001
Mortality at 3months	86 (14.3)	7 (7.2)	0.081

Values are presented as n (%), mean ± standard deviation or median (interquartile range). TIA, transient ischemic attack; eGFR, estimated glomerular filtration rate; NIHSS, National Institutes of Health Stroke Scale; tPA, tissue plasminogen activator; mTICI, modified Thrombolysis in Cerebral Infarction; mRS, modified Rankin Scale. * p-value for trend. 95 patients were assessed with initial eGFR in the excluded patients.

Table S2. Factors associated with functional outcome at 3 months.

	Good outcome (n = 271)	Poor outcome (n = 330)	p-value
Age, years	65.2 ± 12.6	70.2 ± 11.3	<0.001
Sex, men	163 (60.1)	170 (51.5)	0.042
Risk factors			
Hypertension	155 (57.2)	226 (68.5)	0.006
Diabetes mellitus	61 (22.5)	106 (32.1)	0.012
Atrial fibrillation	130 (48.0)	168 (50.9)	0.525
Dyslipidemia	81 (29.9)	99 (30.0)	>0.999
Smoking	60 (22.1)	68 (20.6)	0.721
Previous stroke or history of TIA	45 (16.6)	62 (18.8)	0.556
Medication prior to admission			
Antiplatelets	69 (25.5)	93 (28.2)	0.512
Anticoagulants	40 (14.8)	39 (11.8)	0.347
Statins	11 (4.1)	23 (7.0)	0.174
Baseline renal function			0.005 *
eGFR ≥90 mL/min/1.73 m^2	51 (18.8)	47 (14.2)	
eGFR 60–89 mL/min/1.73 m^2	102 (37.6)	109 (33.0)	
eGFR 30–59 mL/min/1.73 m^2	108 (39.9)	144 (43.6)	
eGFR <30 mL/min/1.73 m^2	10 (3.7)	30 (9.1)	
Presence of acute kidney injury	7 (2.6)	52 (15.8)	<0.001
Acute kidney injury			<0.001 *
No kidney injury	264 (97.4)	278 (84.2)	
Stage 1	4 (1.5)	18 (5.5)	
Stage 2	1 (0.4)	13 (3.9)	
Stage 3	2 (0.7)	21 (6.4)	
Contrast dose, mL	63.1 ± 31.7	77.8 ± 40.0	<0.001
Laboratory findings			
Hemoglobin, g/dL	13.6 ± 1.7	13.3 ± 1.9	0.051
White blood cell count, ×10^9/L	8.3 ± 3.1	9.2 ± 3.8	0.002
Platelets, ×10^9/L	221 ± 69	221 ± 70	0.980
Glucose, mmol/L	7.5 ± 2.9	8.2 ± 3.2	0.004
Stroke-related factors			
NIHSS score on admission	15 (11–19)	19 (15–22)	<0.001
Intravenous tPA	147 (54.2)	163 (49.4)	0.271
Onset to puncture time, min	248 (170–398)	278 (198–480)	0.011
Procedure time, min	50 (38–77.5)	70 (50–104)	<0.001
Unsuccessful reperfusion (mTICI 2a or less)	40 (14.8)	109 (33.0)	<0.001
Outcomes			
Any hemorrhagic transformation	58 (21.4)	142 (43.0)	<0.001
Parenchymal hematoma	16 (5.9)	70 (21.2)	<0.001
Parenchymal hematoma, type 2	6 (2.2)	39 (11.8)	<0.001

Values are presented as n (%), mean ± standard deviation or median (interquartile range). TIA, transient ischemic attack; eGFR, estimated glomerular filtration rate; NIHSS, National Institutes of Health Stroke Scale; tPA, tissue plasminogen activator; mTICI, modified Thrombolysis in Cerebral Infarction. * p-value for trend.

Table S3. Factors associated with mortality at 3 months.

	Alive (n = 515)	Death (n = 86)	p-value
Age, years	67.6 ± 12.2	69.9 ± 11.9	0.103
Sex, men	288 (55.9)	45 (52.3)	0.614
Risk factors			
Hypertension	320 (62.1)	61 (70.9)	0.148
Diabetes mellitus	136 (26.4)	31 (36.0)	0.086
Atrial fibrillation	260 (50.5)	38 (44.2)	0.335
Dyslipidemia	149 (28.9)	31 (36.0)	0.228
Smoking	114 (22.1)	14 (16.3)	0.278
Previous stroke or history of TIA	88 (17.1)	19 (22.1)	0.332
Medication prior to admission			
Antiplatelets	139 (27.0)	23 (26.7)	>0.999
Anticoagulants	68 (13.2)	11 (12.8)	>0.999
Statins	24 (4.7)	10 (11.6)	0.019
Baseline renal function			0.010 *
eGFR ≥90 mL/min/1.73 m^2	88 (17.1)	10 (11.6)	
eGFR 60–89 mL/min/1.73 m^2	187 (36.3)	24 (27.9)	
eGFR 30–59 mL/min/1.73 m^2	210 (40.8)	42 (48.8)	
eGFR <30 mL/min/1.73 m^2	30 (5.8)	10 (11.6)	
Presence of acute kidney injury	30 (5.8)	29 (33.7)	<0.001
Acute kidney injury			<0.001 *
No kidney injury	485 (94.2)	57 (66.3)	
Stage 1	17 (3.3)	5 (5.8)	
Stage 2	5 (1.0)	9 (10.5)	
Stage 3	8 (1.6)	15 (17.4)	
Contrast dose, mL	69.3 ± 37.0	82.1 ± 36.3	0.003
Laboratory findings			
Hemoglobin, g/dL	13.5 ± 1.8	13.2 ± 2.2	0.200
White blood cell count, ×10^9/L	8.7 ± 3.5	9.5 ± 3.8	0.062
Platelets, ×10^9/L	218 ± 67	241 ± 82	0.014
Glucose, mmol/L	7.7 ± 3.0	8.7 ± 3.6	0.016
Stroke-related factors			
NIHSS score on admission	16 (13–21)	19 (16–23)	<0.001
Intravenous tPA	269 (52.2)	41 (47.7)	0.505
Onset to puncture time, min	266 (182–436)	274 (174–434)	0.924
Procedure time, min	60 (42–89)	69.5 (48–140)	0.006
Unsuccessful reperfusion (mTICI 2a or less)	113 (21.9)	36 (41.9)	<0.001
Outcomes			
Any hemorrhagic transformation	161 (31.3)	39 (45.3)	0.015
Parenchymal hematoma	60 (11.7)	26 (30.2)	<0.001
Parenchymal hematoma, type 2	26 (5.1)	19 (22.1)	<0.001

Values are presented as n (%), mean ± standard deviation or median (interquartile range). TIA, transient ischemic attack; eGFR, Estimated glomerular filtration rate; NIHSS, National Institutes of Health Stroke Scale, tPA, tissue plasminogen activator; mTICI, modified Thrombolysis in Cerebral Infarction. * p-value for trend.
